# Effects of Catfish (*Ictalurus punctatus*) Bone Powder on Consumers’ Liking, Emotions, and Purchase Intent of Fried Catfish Strips

**DOI:** 10.3390/foods11040540

**Published:** 2022-02-14

**Authors:** Silvia Murillo, Ryan Ardoin, Evelyn Watts, Witoon Prinyawiwatkul

**Affiliations:** 1 Agricultural Center, School of Nutrition and Food Sciences, Louisiana State University, Baton Rouge, LA 70803, USA; smurillomiguez1@lsu.edu (S.M.); EGWatts@agcenter.lsu.edu (E.W.); 2 USDA-ARS, Southern Regional Research Center, 1100 Robert E. Lee Blvd., New Orleans, LA 70124, USA; Ryan.Ardoin@usda.gov

**Keywords:** catfish, fish bone, bone powder, byproduct, sensory liking, emotions, fried food

## Abstract

Catfish are the predominant U.S. aquacultural product. However, byproducts from filleting, including bones that are high in calcium, typically go to waste or are sold as a low-valued feed. This research evaluated the potential use of catfish bone powder (CBP; 21.07% calcium) as a food ingredient. Catfish fillet strips were dredged with a breading mix (CBPM) containing 0% (0CBPM), 10% (10CBPM), and 20% (20CBPM) CBP before frying. Consumers (N = 211) evaluated sensory liking (nine-point hedonic scale) and attribute intensity (JAR scale), emotions (check-all-that-apply), and purchase intent (PI, yes/no) of samples. Color and texture were measured instrumentally. CBP did not show any negative effects on liking scores, although crispiness was scored higher for 20CBPM (mean = 6.88) than 10CBPM (mean = 6.43). Positive emotions were most relevant to CBP-containing samples, with significantly higher rates of *adventurous* and *understanding*. Information about calcium fortification using CBP increased PI to 81.04% for the 10CBPM and 83.89% for the 20CBPM samples and showed a greater effect on Latin Americans/Hispanics than U.S. Americans. Consumers were not averse to the consumption of CBP which can contribute to sustainable nutrition through waste reduction. Successful calcium fortification of fried catfish dredged with 20% CBP did not compromise sensory liking and may be feasible in other products.

## 1. Introduction

Globally, the rate of increase in fish consumption has been outpacing that of terrestrial proteins and the human population growth rate [1]. Of the approximately 179 million tons of fish produced yearly, around 80% is intended for human food, and about one-third of that becomes loss or waste. Fish waste is disproportionately high in North America [1], and in the U.S., catfish species are the leading aquacultural product [2]. Therefore, developing new commercially viable food applications for would-be catfish waste (i.e., processing byproducts) may reduce waste from the seafood/aquaculture sector and increase profitability for catfish producers while providing additional nutrient sources to the food industry.

The major commercial outputs of catfish processing include fillets, shank fillets, strips and nuggets [3]. The remaining viscera, heads, skin and frames are considered byproducts [4]. Byproducts can account for over 60% of the whole fish weight, and the frame which remains after filleting (backbone and ribs with some muscle, fat and nerve tissues still attached) comprises around 18% of the original catfish mass [3]. As the primary component of frames, fish bones can be used as a low-cost source of calcium and phosphorous in human food [5].

Calcium (Ca) is an essential micronutrient critical to bone health, blood clotting, nerve transmission and muscle function. Inadequate Ca consumption is associated with an increased risk of osteoporosis, hypertension and colon cancer. The Recommended Dietary Allowance (RDA) for Ca is 1000 mg/day for 19–51 years old and 1200 mg/day for older adults, but the average global intake is only 400 mg/day and less than 300 mg/day in undeveloped countries [6,7]. This current research proposes that acceptable food-based applications of catfish bone can contribute to the variety of options available for adequate calcium uptake.

While the present research utilized catfish (*Ictalurus punctatus*) frames, value-added processing of bones from other freshwater [8,9] and saltwater [10] fish species have been studied. By incorporating safe-to-eat fish bone powders into food products, the calcium content of foods such as cookies [8,11], bread [5], fish sausage [12], noodles [13] and surimi [14] was increased. Using Nile tilapia bone powder, for example, a 12% replacement of wheat flour in flaxseed cinnamon cookies [8] and a 20% replacement of wheat flour in cashew nut cookies [11] provided calcium fortification without compromising product acceptability. However, the level of bone powder usage deemed acceptable by consumers varies between product-type and population segment, and consumer perceptions of edible fish bone powder in the U.S. have not been fully explored.

U.S. consumers are increasingly concerned with the source of ingredients in their foods [15]. As such, information pertaining to byproducts’ origin and/or potential health benefits can increase the purchase intent of products containing them, and relevant food-evoked emotions can enrich understanding of product differences [16]. In both the U.S. and European markets, the demand for “natural” food additives is seen as a current and ongoing trend [17]. Although there is no single objective definition, consumers’ expectations of naturalness may be met by a calcium source that is familiar in origin (i.e., fish) and produced without synthetic chemical usage [15]. Effective marketing strategies for calcium-fortified foods in a particular context should be cost-effective and consider consumers’ attitudes as well as cultural acceptability [18].

The objectives of this study were to evaluate U.S. consumers’ liking, emotional profiles, and purchase intent (PI) associated with fried catfish strips dredged with catfish (*Ictalurus punctatus*) bone powder breading mixes. Catfish bone powder (CBP) was produced using a simple and inexpensive method without chemical treatment, and its mineral content along with selected physical properties (color and texture) of fried catfish strips were also analyzed. Additionally, the impact of a bone powder information message (BPI) on PI was investigated. 

## 2. Materials and Methods

### 2.1. Preparation of CBP

Channel catfish (*Ictalurus punctatus*) frames were obtained from a local processor (Breaux Bridge, LA, USA) as a byproduct of filleting. Length of frames (with heads removed) ranged from 50 cm to 60 cm, and frame weight was between 0.64 kg and 0.77 kg. Frames were transported on ice, stored at −20 °C for approximately 48 h, then thawed overnight at 2 °C before being processed into CBP. While similar procedures were used elsewhere for fish bone powder production, the following processing parameters were developed specifically for this study based on the nature of the raw material. To produce CBP, frames were first boiled for 10 min, then manually scrubbed with a brush to remove flesh and rinsed with tap water. This process was repeated. The cleaned frames were oven-dried (OV310G rotating rack oven, BAXTER Inc., Deerfield, IL, USA) at 105 °C for 6 h. Dried frames were then ground for 30 s in a commercial grinder (CGOLDENWALL Inc., Hangzhou, China). The ground bone was sieved to recover only particles 1.0 mm or smaller. The resultant CBP was stored in sealed plastic zip-bags under refrigeration (2 °C) for 3 days prior to incorporation into a breading mix and catfish strip preparation.

### 2.2. Mineral Analysis of CBP

The mineral and metal composition of CBP was analyzed in quadruplicate at the Louisiana State University AgCenter Agricultural Chemistry laboratory (Baton Rouge, LA, USA), which is ISO 17025 accredited. Each CBP sample (0.49 to 0.50 g) was placed into a 55-mL PFA microwave digestion tube (CEM, Matthews, NC, USA). A total of 12 mL concentrated acid was added to each tube (10 mL of trace metal grade nitric acid and 2 mL of trace metal grade hydrochloric acid). The tubes were capped and digested in a microwave digestor (MARSXpress, CEM, Matthews, NC, USA). The digestor was ramped for 20 min at 1600 W to 200 °C and held at this temperature and power for 15 min, followed by a 15 min cooldown. Samples were transferred into acid-washed class A 100 mL volumetric flasks and diluted with deionized water after the microwave digestion and cooldown were completed. All samples were analyzed by inductively coupled plasma-optical emission spectroscopy (ICP-OES; Optima 8300, Perkin Elmer, Inc., Waltham, WA, USA). 

Samples were tested using a modified AOAC Method 985.01 [19]. A total of 21 minerals and metals were evaluated: boron (B), calcium (Ca), copper (Cu), iron (Fe), magnesium (Mg), manganese (Mn), phosphorus (P), potassium (K), sodium (Na), sulfur (S), zinc (Zn), aluminum (Al), barium (Ba), cadmium (Cd), chromium (Cr), cobalt (Co), lead (Pb), molybdenum (Mo), nickel (Ni), selenium (Se) and arsenic (As). Mineral and metal concentrations were reported in parts per million (ppm), parts per billion (ppb) or percentage (%) on a dry weight basis, as shown in Table 1. 

Mercury (Hg) concentration in CBP was analyzed in quadruplicate using a Milestone Direct Mercury Analyzer 80 (DMA80, Milestone Inc., Sorisole, Italy). The DMA80 methodologies used followed the EPA method 7473 [20] and ASTM D-6722-01 method [21]. The DMA80 detects total mercury (organic and inorganic) by thermal decomposition, amalgamation, and atomic absorption spectrophotometry. The typical working range for this instrumentation is 0.05–300 ng with instrument detection limits at 0.01 ng. Samples were weighed (0.05 ± 0.002 g) into metal weighing boats and placed in the DMA80. The absorbance peak areas were measured at 253.7 nm, and results were reported in parts per billion (ppb).

### 2.3. Catfish Strip Preparation

Fried catfish strips were chosen as a carrier for CBP addition due to regional familiarity and preliminary data on product appropriateness, rather than for healthfulness. CBP breading mixes (CBPM) were prepared using a commercial seafood breading mix (yellow corn meal, yellow corn flour, salt, monosodium glutamate, spices, citric acid, dried garlic, paprika, calcium phosphate; Louisiana Fish Fry Products Ltd., Baton Rouge, LA, USA) with incorporation of CBP at 0 (0CBPM), 10 (10CBPM) and 20 (20CBPM) percent by weight of the final mix. Fresh catfish fillets (7–9 ounces each) were obtained from a local processor (Breaux Bridge, LA, USA). Fillets were cut into uniform rectangular strips (length × width × thickness of 7 cm × 2 cm × 1.5 cm), and strips were dredged with the CBP breading mix (0CBPM, 10CBPM or 20CBPM). Dredged strips were fried in canola oil (Wesson Pure Canola Oil; Bentonville, AR, USA) for 4 min at 190 °C in an electric deep frier (Hamilton Beach 3-qt. Deep Fryer, Reno, NV, USA). Freshly fried samples were held in a 170 °F (79.4 °C) oven before serving, and were replaced with freshly fried samples every 20 min.

### 2.4. Color and Texture Analysis

Color and texture of fried catfish strips were measured (10 replications per treatment). Surface color of strips was measured as L*, a*, b* values using a colorimeter (BC-10, Konica Minolta, Inc., Osaka, Japan). The delta-E (∆E) pairwise color difference was calculated (Equation (1); L*, a* and b* represented mean values of each respective index, and subscripts 1 and 2 referred to two samples of interest; [22].

(1) $$\Delta E=\sqrt{{\left(L_{2}^{*}-{\;L}_{1}^{*}\right)}^{2}+{\left(a_{2}^{*}-{\;a}_{1}^{*}\right)}^{2}+{\left(b_{2}^{*}-{\;b}_{1}^{*}\right)}^{2}}$$

For texture analysis, fried strip portions (3 cm × 2 cm × 2 cm pieces) that were cooled to room temperature were measured (10 replications per treatment) with a texture analyzer (TA-XTPlus, Texture Technologies, Godalming, UK) via two compressions with a two-inch diameter cylindrical aluminum probe (TA-25, Texture Technologies). Samples were compressed by 30% (pre-test speed of 2 mm/s, test speed of 1 mm/s, and post-test speed of 5 mm/s). As the more relevant TPA parameters for the current product, hardness (N) and cohesiveness were reported.

### 2.5. Consumer Test

This research involving human subjects was approved by the Louisiana State University (LSU) Agricultural Center Institutional Review Board (IRBAG-21-0063). The consumer study was conducted at the LSU AgCenter Sensory Services laboratory (Baton Rouge, LA, USA) in partitioned booths. A total of two hundred eleven adults (N = 211 consumers at least 18 years of age) were recruited from LSU campus and screened for relevant allergens to participate in the consumer test. To be selected for participation, panelists must generally consume fish and fish products. The consumer sample was comprised of 52% males and 48% females, with a majority being U.S. American (52%) or Latin American/Hispanic (25%) and between the ages of 18–25 (62%) or 26–35 (29%). Panelists were not compensated for participation. 

Each consumer evaluated three total fried catfish strips (0CBPM, 10CBPM and 20CBPM) following a balanced and randomized complete block design. Samples were labeled with three-digit blinding codes. Unsalted crackers and water were used for palate cleansing. 

Data were collected via an online questionnaire (Qualtrics software, Provo, UT, USA). Consumers rated samples for liking of overall visual quality, surface color and aroma before tasting, followed by liking of overall texture, flavor and overall liking after tasting (labeled 9-point hedonic scale, anchored at dislike extremely = 1, neither like nor dislike = 5 and like extremely = 9). Brownish color and crispiness of fried catfish strips were rated using a 3-point Just-About-Right (JAR) scale (not enough/JAR/too much). After hedonic and JAR scoring, purchase intent (PI; yes/no) of each sample was reported before and after presentation of the following bone power information message (BPI) for 10CBPM and 20CBPM only: “The breading mix was supplemented with edible and safe fish bone powder to increase calcium, which may provide health benefit”. Lastly (after BPI presentation for 10CBPM and 20CBPM only), consumers reported emotional responses to samples from a list of 25 terms in a check-all-that-apply (CATA) format. The emotion list used was the EsSense25 lexicon [23], modified by replacing secure with unsafe [24].

### 2.6. Statistical Analysis

Data were analyzed using SAS software (Copyright© 2016 SAS Institute Inc., Cary, NC, USA). Multivariate analysis of variance (MANOVA) was used to test for overall differences in liking among fried catfish strip samples, followed by descriptive discriminant analysis (DDA) [25] and univariate analysis of variance (ANOVA, with Tukey’s post-hoc test) to explore differentiating sensory attributes. ANOVA was also used to analyze instrumental color and texture measurements of fried catfish strips. JAR data were used to conduct penalty analysis [26,27] to determine the effects of non-JAR responses for crispiness on overall liking and liking of overall texture and crispiness. Logistic regression analysis (LRA) was used to identify significant predictors of PI, both before and after the BPI was given to consumers. Cochran’s Q test was used to test for differences in PI and emotions across samples. McNemar’s test for marginal homogeneity investigated changes in PI frequency after delivery of BPI.

## 3. Results and Discussion

### 3.1. Mineral Content of Catfish Bone Powder

Along with species, processing methods have been shown to influence the composition of fish bone powder [28,29]. Bechtel et al. [4] reported channel catfish frames to contain around 20% lipid and 16% protein. As an alkaline boiling media can denature and hydrolyze proteins and saponify fats [29], its use for fish bone powder production has been commonly employed, often followed by acid treatment to neutralize the bone mixture [5,10,11]. Others have tested the ability of ultrasonic irradiation [30], proteolytic enzymes and high-pressure wash [3] to remove excess material from frames. Chemicals such as hexane [10], bleach [11], hydrogen peroxide and organic acids [9] were also used in processing in an attempt to optimize fishbone quality. Compared to the aforementioned studies, the method of CBP production in the present study was simpler without chemical treatment, and, therefore, a potentially greener and less expensive technology.

The resultant CBP produced for this experiment contained 21.07% Ca and 9.14% P by weight (dry wt basis, Table 1). Using only boiling water as a solvent, Bechtel et al. [3] found 21.27% Ca and 9.85% P (dry wt basis) in channel catfish bone fractions. Other researchers have reported 18.07% Ca and 8.83% P in hake bone powder [31] and 23.69% Ca in silver carp bone powder [32] using similar methods. Alkaline treatments, however, have consistently yielded fish bone powders with higher proportions of calcium and phosphorus: 30.67% Ca and 16.65% P from seabream [28], 32% Ca from silver carp [12] and 38.16% Ca and 23.31% P from tuna [5]. Despite lower levels than would be expected from chemical processes, the presently observed Ca/P ratio in CBP of 2.3/1 is similar to that found in human bones [33], and a Ca/P ratio above one is ideal for Ca bioavailability [34]. Additionally, the content of heavy metals in CBP (Table 1) was below the general recommended limits in additives of 2 mg/kg for lead, 1 mg/kg for cadmium and 1 mg/kg for mercury [35].

To meet the RDI of 1000 mg Ca from CBP alone would require consumption of 4.72 g (dry wt basis) CBP daily, equivalent to 25 g of 20CBPM. CBP and subsequent food applications are not intended to be a sole source of dietary calcium, but rather to provide additional options as part of a whole-diet approach to health [36]. Fortification of foods with Ca is one strategy to address dietary calcium deficiencies [6,7]. In particular, consumers with intolerance or allergy to dairy (which accounts for 72% of U.S. Ca intake) may benefit from more food-based alternatives to Ca supplementation [7]. In countries where dairy intake is low and osteoporosis is more prevalent, approaches to Ca fortification must be economically sustainable [37], and utilization of CBP presents an option for fortification of non-staple foods. 

### 3.2. Overall Product Differences Based on Liking of Catfish Strips Dredged with CBP Breading Mixes

The appeal of fried food relies on a sensory experience involving appearance, aroma, texture and flavor [38]. Frying is a popular cooking method for catfish, and fried fish is commonly consumed in Southern parts of the U.S. [39]. From the present population sample, 76% of the N = 211 consumers reported prior consumption of fried catfish strips.

Consumers rated catfish strips similarly and favorably across treatments and attributes, with mean liking scores ranging from 6.43 (for surface crispiness of 10CBPM) to 7.23 (for the overall visual quality of 10CBPM; Table 2). MANOVA provided some evidence for an overall difference in hedonic quality among catfish strips dredged with different breading mixes, albeit with a Wilk’s Lambda test *p*-value = 0.057. Univariate ANOVAs were performed as follow-up tests [25]. Therefore, we must acknowledge that the overall type I error rate for these tests may be as high as 0.057. 

Comparing individual sensory attributes between treatments, no significant differences (*p*-values > 0.05) were found between catfish strips dredged with a commercial breading mix only (0CBPM) and those dredged with 10CBPM or 20CBPM (Table 2). The 20CBPM strips were rated directionally highest in overall liking (mean score of 7.00, or “like moderately” on the labeled nine-point scale) and PI (83.9% “yes” after BPI; Table 2). Collectively, these data indicated that at 10% and 20% CBP addition levels in breading mixes, CBP did not compromise the sensory quality of fried catfish strips. This result is encouraging for CBP utilization in the current food matrix, as successful Ca fortification should not cause changes to sensory properties of the final product [37]. However, the observed outcomes within fried fish breading are not necessarily generalizable to other product applications. Although no negative effects on the aroma or flavor liking of fried catfish were observed (Table 2), other authors have described a heavy fish odor from bone powders produced without chemical treatment [28], which may be undesirable in some products. Njoroge and Lokuruka [9] processed tilapia frames with citric acid to remove fishy aroma before incorporation into cookies. Depending on formulation parameters, milkfish bone powder imparted a slightly perceptible fish flavor to noodles with 1% incorporation, and a moderately perceptible fish flavor at 10% [13]. In the present study, any fish aroma or flavor inherent to CBP may have been indistinguishable from that of the catfish flesh or commensurate with expectations of fried fish.

### 3.3. Color of Fried Catfish Strips Dredged with CBP Breading Mixes

The desired color of battered fried foods is golden-brown [40]. When asked to rate the brown color of fried catfish samples (JAR scale), 79.6%, 80.1% and 78.2% of consumers rated it as “just about right” for strips dredged with 0CBPM, 10CBPM, and 20CBPM, respectively. Although surface color measurements of 20CBPM strips showed statistically lower a* values (less redness; *p*-value < 0.05; Table 2) than those with less or no CBP, all calculated pairwise ∆E values were less than the just noticeable difference of ∆E ≈ 2.3 [22] which approximates the color difference discernable by the human eye. The mean surface color liking scores among fried catfish samples were favorable (all above seven or “like moderately” on the nine-point scale; Table 2) and not significantly different (*p*-value > 0.05). 

### 3.4. Texture of Fried Catfish Strips Dredged with CBP Breading Mixes

Samples were liked similarly in terms of overall texture (6.54–6.85, Table 2), but directing consumers’ attention to the specific dimension of surface crispiness provided additional information on the effects of CBP in breading mixes. Of all sensory attributes evaluated, only liking of surface crispiness significantly differed (*p*-value = 0.038) between 10CBPM (mean score = 6.43) and 20CBPM (mean score = 6.88). Accordingly, crispiness liking was the most discriminating hedonic attribute among treatments based on DDA, with the highest pooled within the canonical correlation of 0.61 in Can1, which accounted for 75% of the total explained variance (Table 3). 

**Figure 1:**
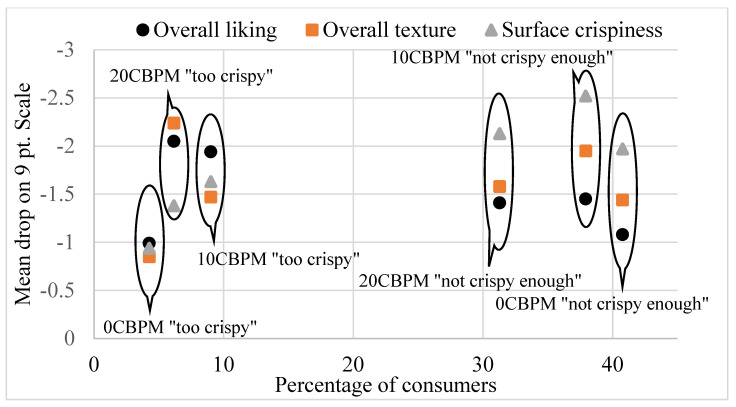
Crispiness penalty plot of fried catfish strips based on a 3-point JAR scale (not crispy enough, JAR, too crispy); 0CBPM, 10CBPM and 20 CBPM = breading mix with 0%, 10% and 20% CBP, respectively.

Crispy texture is a critical characteristic of deep-fried foods and contributes to their palatability [38]. In this study, liking scores for surface crispiness were more heavily penalized when perceived to be “not crispy enough” than when “too crispy”. “Not crispy enough” response rates ranged from 31.3% (20CBPM) to 40.8% (0CBPM), resulting in concerning penalties [26] on surface crispiness liking of 1.97 (for 0CBPM), 2.13 (for 20CBPM) and 2.52 (for 10CBPM) units on the nine-point scale (Figure 1). Though samples were rated as “too crispy” by less than 10% of consumers (Figure 1), mean OL scores dropped by around two units for 10CBPM (mean-drop of 1.94) and 20CBPM (mean-drop of 2.05) for this category. 

The crispiness is characterized by the ease and speed of fracturing a rigid food (related to force) as well as the volume and frequency of sounds emitted [41]. Instrumental hardness, recorded as the maximum force to deform fried catfish strips, did not significantly differ across samples (Table 2), although that of 10CBPM was slightly lower (5.98 N vs. 6.55–6.61 N). Likewise, cohesiveness was significantly lower (*p* < 0.05) for strips dredged with 10CBPM than both other treatments. That is, the fried catfish portions dredged with 10% CBP in the mixture did not hold together from the first compression to the second as well as the others. Based on definitions from Shih et al. [40], instrumental crispiness and cohesiveness are inversely related in that more cohesive samples deform without fracturing or breaking. Comparing samples containing 10% and 20% CBP, it was inferred from the relative proportion of non-JAR responses and associated mean-drops that being “not crispy enough” had a greater negative impact on the surface crispiness liking scores for 10CBPM. A possible explanation for the observed difference in surface crispiness liking between 10CBPM and 20CBPM (Table 2) is a potentially bimodal distribution of consumers who either favored the crispiness of boneless breading mix or that with a higher CBP (i.e., 20%). Although the magnitude of the difference was not statistically significant at α = 0.05, 0CBPM did have directionally higher mean crispiness liking than CBPM10 (6.60 vs. 6.43; Table 2) and a slightly higher proportion of JAR responses (55% vs. 53%) compared to 10CBPM. For 20CBPM, crispiness was considered JAR by 63% of consumers.

### 3.5. Emotional Responses Elicited by Fried Catfish Strips Dredged with CBP Breading Mixes

Food-evoked emotions have been shown to differentiate food products and predict purchase intent better than liking scores alone [24,42]. Emotional responses may be particularly relevant to understanding consumers’ willingness to consume ingredients from novel sources such as catfish byproducts. After evaluating their sensory characteristics and receiving the bone powder information message (BPI; for 10CBPM and 20CBPM only), consumers were asked how they felt about consuming fried catfish samples. A 20% selection rate was used to distinguish the most salient food-evoked emotions associated with a given product [43]. By this criterion, emotions most relevant to fried catfish strips dredged with CBP breading mix were generally positive: *active*, *calm*, *enthusiastic*, *good*, *happy*, *interested*, *joyful*, *mild*, *pleasant*, *satisfied*, *understanding* and *warm* (Table 4). Notably, none were negative, although the *bored* emotion for 0CBPM approached 20%. The valence of *mild* and *understanding* are unclear [43].

Positive emotions *good*, *satisfied*, *pleasant*, *happy*, and *interested* were associated with CBP-containing samples by 45–60% of consumers, followed by *calm* with 30%–40% of consumers. The most prevalent negative emotion was *bored*, being selected by 17% of consumers after consuming bone-free 0CBPM samples, for which no informational cue (i.e., BPI) was given. Low selection rates (<8% of consumers) of *worried*, *unsafe*, *disgusted* and *guilty* lent credence to consumers’ openness to consumption of CBP delivered in a familiar and readily acceptable product. This result may suggest that most consumers were not concerned with the safety or origin of CBP.

While liking scores were able to best discriminate samples in terms of crispiness, emotions *adventurous* and *understanding* also differed between samples (Cochran’s Q test; Table 4), providing greater resolution to perceptual differences. As catfish bone is not typically consumed in the U.S., higher rates of *adventurous* (approximately 19% for 10CBPM and 20CBPM compared to 10% for 0CBPM; Table 4) may have been related to increased arousal and positive regard for new eating experiences [44]. The term *understanding* was also reported more frequently for samples containing CBP (17.5%–20.4% vs. 11.4%). It is proposed that these responses were attributable to consumers’ enhanced intellectual “understanding” of the CBP-containing products via BPI, rather than the sympathetic emotion of understanding. Similar explanations were offered for prevalent associations of *mild* and *warm* with foods for which it was unclear whether emotion terms related to characteristics of the stimulus or feelings evoked by it [44]. In the development of the EsSense Profile™ [43], consumers did not consistently agree on whether *understanding* was positive, negative, neither or both. However, by making consumers aware of CBP usage and the potential health benefit, and comparing samples to the control (0CBPM), it was evident that emotive responses to the sensorial (fried catfish strips) and informational (BPI) stimuli were generally favorable.

### 3.6. Predicting Purchase Intent of Fried Catfish Strips as Affected by a Bone Powder Information (BPI) Message

Proportions of positive PI (“yes” responses) were similarly high for 10CBPM (71.56%), 20CBPM (73.93%) and the control product 0CBPM (71.09%; Table 2). After presentation of BPI, positive PI increased significantly (*p*-value < 0.05) to 81.04% for 10CBPM and 83.89% for 20CBPM. Depending on consumers’ attitudes toward the nature of an ingredient, the source information may improve product acceptability [15] or dimmish it [44]. The impact of health benefit information can also depend on intrinsic sensory characteristics of the product itself (e.g., color and flavor) [16]. It is hypothesized that favorable sensory quality and positive reactions to the Ca source (fish bone) were moderators of the significant positive effect that BPI had on PI of 10CBPM and 20CBPM.

Logistic regression analysis (LRA) was used to model PI odds based on gender, ethnicity/nationality and each dimension of sensory liking (overall visual quality, surface color, aroma, overall texture, surface crispiness and flavor), both before and after the BPI was given to consumers (Table 5). When health benefit claims are impactful and PI response rates change based on new information, so can the set of significant predictors used to model these responses [42]. That is, some variables which were significant to the initial PI response, may not be significant in predicting the new response which is based on additional information, and vice versa. 

Before BPI was given to consumers, liking of overall texture, flavor and surface crispiness showed a significant relationship with PI odds (Table 5). Based on odds ratio (OR) estimates, odds of positive PI would be expected to increase by 39%, 62% and 117% for every one-unit increase in liking of overall texture (OR = 1.39), surface crispiness (OR = 1.62) and flavor (OR = 2.17), respectively. On the labeled nine-point hedonic scale this would equate to comparing PI odds from any one ordered hedonic category to the next, e.g., when the attribute is rated as six = “like slightly” vs. seven = “like moderately.” Of these three sensory attributes, flavor scores exhibited the least amount of variability both across and within samples but were most impactful on the PI. 

For PI responses after delivery of the BPI, only liking of flavor remained a significant predictor (OR = 1.53). However, a significant effect of ethnicity/nationality was revealed, where expected odds of positive PI for Latin American or Hispanic consumers were over twice that of U.S. American consumers, holding all other variables constant (OR = 2.51; Table 5). Consulting the raw data, more Latin Americans/Hispanics were swayed toward purchase than were U.S. Americans by the BPI. In fact, a few U.S. consumers were influenced negatively by the BPI (“yes” before and “no” after), whereas all changes in PI responses from Latin American/Hispanic consumers were from “no” before the BPI to “yes” after the BPI. This analysis demonstrated a cross-cultural difference between these two groups in responsiveness to product information for fried catfish strips, as opposed to differences in sensory preferences [45]. It should be mentioned that these interpretations should be restricted to the present population sample, which was largely comprised of college students. 

The consistent and relatively large impact of flavor liking on PI was not surprising, as taste and flavor are among the strongest drivers of food choice [46], and so was the initial importance of texture and crispiness due to quality expectations of fried foods [38]. After the BPI, however, texture and crispiness liking were no longer significant predictors of the PI response. We suspected that as a result of the BPI, a shift in consumers’ expectations of texture occurred for fried fish samples, perhaps due to the previously discussed levels of “understanding” about bone powder usage. It is possible that some consumers who were initially dissuaded from PI by non-JAR textural properties were willing to change their responses due to new information about potential health benefits.

### 3.7. Limitations

Interpretation of the results reported in this study should be limited to the consumer sample, food matrix, and CBP preparation method under investigation. Catfish has been considered a regional food in the U.S. states where this study was conducted [39], and so attitudes towards CBP, that is catfish byproduct, may not be generalized to different regions or cultures. Potential allergenicity may be a disadvantage of CBP’s use for broader calcium fortification applications. Since the current carrier for CBP application was fried catfish strips, and consumers were warned of fish allergen prior to participation, the allergenicity of CPB was not investigated. As fishy odors and flavors were noted in other fish bone powders [9,13,28], acceptable application of CBP to non-fish food matrices will also likely depend on the sensory properties of the CBP itself. Therefore, descriptive sensory analysis of CBP would be beneficial to understanding its sensory properties. Other limitations of CBP use in varied food products may relate to its functional properties, though these may be enhanced through non-chemical processing methods [14]. Therefore, the suitability of CBP for other products and broader calcium fortification applications should be explored with the specific consumer segment, final product quality, and processing requirements in mind.

## 4. Conclusions

Through relatively simple and inexpensive processing methods, channel catfish frames yielded CBP with over 20% calcium. This CBP was successfully incorporated into a breading mix by up to 20% addition by weight, without hindering the sensory quality of the final product. Beyond sensory quality, psychological associations (i.e., emotions and reactions to product knowledge) with CBP in fried catfish strips also seemed positive. The impact of bone powder information was particularly relevant to Hispanic college students. The potential for calcium fortification in foods using fish bone presents opportunities for processors to reduce waste and add value to byproducts. While the current results are not yet generalizable to other food products, they do promote further investigation of fish bone powder usage and consumer perceptions thereof.

## Figures and Tables

**Table 1:** Mineral and metal composition^^^ of catfish bone powder (dry wt basis).

Analyte	Concentration	Units
Boron	16.00 ± 0.0	PPM
Calcium	21.07 ± 0.6	%
Cooper	4.00 ± 0.0	PPM
Iron	7.78 ± 0.6	PPM
Magnesium	0.35 ± 0.0	%
Manganese	35.48 ± 0.5	PPM
Phosphorus	9.14 ± 0.2	%
Potassium	0.19 ± 0.0	%
Sodium	0.44 ± 0.0	%
Sulphur	0.16 ± 0.0	%
Zinc	118.79 ± 2.5	PPM
Aluminum	31.55 ± 4.7	PPM
Barium	10.90 ± 2.7	PPM
Cadmium	0.20 ± 0.0	PPM
Chromium	4.20 ± 0.1	PPM
Cobalt	0.20 ± 0.0	PPM
Lead	1.20 ± 0.0	PPM
Molybdenum	0.80 ± 0.0	PPM
Nickel	1.22 ± 0.2	PPM
Selenium	16.00 ± 0.0	PPM
Arsenic	4.00 ± 0.0	PPM
Mercury	10.71 ± 3.8	PPB

^^^ Reported as mean ± standard deviation from quadruplications.

**Table 2:** Liking ^1^, purchase intent ^2^, and instrumental color and texture ^3^ of fried catfish strips dredged with CBP breading mixes (CBPM).

Attributes	CBPM0	CBPM10	CBPM20
Visual Quality	7.12 ± 1.3	7.23 ± 1.3	6.97 ± 1.5
Surface Color	7.20 ± 1.4	7.22 ± 1.3	7.12 ± 1.4
Aroma	6.97 ± 1.3	7.12 ± 1.4	7.10 ± 1.4
Surface Crispiness	6.60 ±1.7 ^ab^	6.43 ± 1.9 ^b^	6.88 ± 1.7 ^a^
Overall Texture	6.57 ± 1.6	6.54 ± 1.7	6.85 ± 1.6
Flavor	6.96 ± 1.6	6.94 ± 1.6	7.02 ± 1.6
Overall Liking	6.80 ± 1.5	6.87 ± 1.5	7.00 ± 1.5
**Purchase Intent** (%) Before	71.09	71.56 ^B^	73.93 ^B^
After	-	81.04 ^A^	83.89 ^A^
Color			
L*	58.82 ± 2.0	60.20 ± 3.1	58.87 ± 1.6
a*	8.62 ± 1.4 ^b^	8.62 ± 1.9 ^b^	7.21 ± 0.6 ^a^
b*	24.11 ± 1.9	23.61 ± 2.9	24.30 ± 1.9
**Texture**			
Hardness (N)	6.55 ± 1.3	5.98 ± 1.0	6.61 ± 1.5
Cohesiveness	0.64 ± 0.0 ^a^	0.60 ± 0.1 ^b^	0.66 ± 0.0 ^a^

^1^ Reported as mean ± standard deviation from 211 consumer responses (9-point hedonic scale). ^a,b^ Values in the same row followed by different lowercase letters are significantly different (ANOVA with Tukey’s post-hoc test, *p*-value < 0.05). ^2^ Reported as percentage of “yes” responses from 211 consumers (a yes/no scale), “Before” and “After” a bone powder information (BPI) message. ^A,B^ Values in the same column followed by different capitalized letters are significantly different (McNemar’s test, *p*-value < 0.05). ^3^ Instrumental Color and Texture values are reported as mean ± standard deviation from 10 replications. Values in the same row followed by different letters are significantly different (ANOVA with Tukey’s post-hoc test, *p*-value < 0.05).

**Table 3:** Pooled within canonical correlation values ^1^ describing group differences among fried catfish strips.

Attribute	Can1	Can2
OVQ	−0.47	0.05
Color	−0.19	−0.01
Aroma	0.003	0.57
Crispiness	0.61	0.09
Texture	0.47	0.31
Flavor	0.12	0.09
OL	0.24	0.45
Variance explained (%)	75.5	24.5
Pr > F	0.057	0.455

^1^ Based on Descriptive Discriminant Analysis using liking scores (9-point hedonic scale). Can1 and Can2 refer to the first and second canonical dimensions, respectively.

**Table 4:** Consumer emotional responses (%) ^1^ elicited by fried catfish strips ^2^.

Emotion	0CBPM	10CBPM	20CBPM
Active	23.7	21.3	22.3
Adventurous	10.4 ^b^	18.9 ^a^	19.4 ^a^
Aggressive	3.3	2.4	5.7
Bored	17.16	12.8	10.9
Calm	39.3	40.3	30.3
Disgusted	5.7	8.1	7.6
Enthusiastic	18.0	18.9	20.4
Free	13.7	17.1	19.9
Good	57.8	61.6	60.2
Good-natured	15.6	19.4	19.9
Guilty	3.8	1.9	1.4
Happy	49.3	45.9	49.3
Interested	46.5	52.6	54.5
Joyful	21.3	19.9	26.5
Loving	10.4	9.5	9.9
Mild	26.5	30.3	24.6
Nostalgic	13.7	12.3	12.8
Pleasant	45.9	49.4	51.7
Satisfied	54.5	52.6	51.2
Unsafe	4.3	2.8	4.3
Tame	16.1	11.9	9.9
Understanding	11.4 ^b^	20.4 ^a^	17.5 ^a^
Warm	29.9	24.6	26.1
Wild	7.6	5.7	7.6
Worried	6.2	6.2	4.7

^1^ Percentage of N = 211 consumers who selected each emotion from a CATA list. ^a,b^ Values in the same row followed by different letters indicate significant differences in selection rates (Cochran’s Q test, Bonferroni adjusted *p*-value < 0.0167). ^2^ 0CBPM, 10CBPM and 20 CBPM = breading mix with 0%, 10% and 20% CBP, respectively.

**Table 5:** Odds ratio estimates ^1^ for predicting purchase intent (PI) based on logistic regression modeling.

Response	PI before ^2^		PI after ^3^	
	Pr > ChiSq	Odds Ratio	Pr > ChiSq	Odds Ratio
Gender	1.16	1.42	0.80	1.08
Nationality ^4^	0.70	-	**0.001**	**2.51**
Overall Visual Quality	0.95	1.00	0.75	0.95
Color	0.97	1.01	0.25	1.23
Aroma	0.14	0.85	0.25	0.87
Texture	**0.005**	**1.39**	0.08	1.29
Flavor	**<0.0001**	**2.17**	**0.0002**	**1.53**
Crispiness	**<0.0001**	**1.62**	0.10	1.22

^1^ Significant odds ratios are in bold typeface (*p*-value < 0.05). ^2^ Odds of PI before presentation of a bone powder information (BPI) message. ^3^ Odds of PI after presentation of a BPI message. ^4^ The nominal variable Nationality had 7 levels. There was no significant effect of Nationality on “PI before”. For “PI after”, odds were significantly higher for Latin American/Hispanic than U.S. American consumers.

## Data Availability

The data that support the findings of this study are available from the corresponding author upon reasonable request.
